# Percutaneous intestinal drainage for a refractory enterocutaneous fistula: A case report

**DOI:** 10.1016/j.ijscr.2020.06.089

**Published:** 2020-06-25

**Authors:** Yoshie Hirayama, Tadashi Koga, Masanori Kai, Kiyoshi Kajiyama

**Affiliations:** Dept. of Surgery, Iizuka Hospital, 3-83 Yoshiomachi, Iizuka City, Fukuoka, 820-8505, Japan

**Keywords:** Refractory enterocutaneous fistula, Treatment, Percutaneous drainage

## Abstract

**Introduction:**

Enterocutaneous fistulas (ECFs) that occur following gastrointestinal surgery require long-term hospitalization, and treatment may be difficult in rare cases. Although the morbidity and mortality associated with ECF have decreased with modern medical, the overall mortality is still surprisingly high, up to 30.4 %.

**Presentation of case:**

The patient was a 79-year-old male who had undergone laparoscopic sigmoidoscopy for sigmoid colon cancer 5 years previously. He was newly diagnosed with sigmoid colon cancer 5 years following surgery. A laparoscopic high anterior resection was performed. On the 4th postoperative day, he was diagnosed with a suture failure which was treated conservatively; however, the fistula could not be closed, and ileostomy construction was performed. Intestinal fluid leaked from the median surgical incision, leading to the formation of a small intestinal fistula on the proximal side from the ileostomy. Conservative treatment did not improve the condition and skin erosion worsened. Two months after the stoma was constructed, a urethral balloon catheter was percutaneously inserted into the intestinal tract from the small intestinal fistula to drain the intestinal fluid. Following the maneuver, the problem of skin erosion was improved, with the resulting closure of the fistula.

**Discussion:**

The basic principles underlying treatment for ECFs are essentially fasting, drainage, and adequate nutritional management. Some studies reported that the average period of negative pressure therapy was four weeks. It seems that four weeks is the breakpoint.

**Conclusion:**

Percutaneous intestinal drainage for refractory ECFs following gastrointestinal surgery is minimally invasive and is likely to be extremely useful.

## Highlights

• Treatment for enterocutaneous fistulas are essentially fasting, drainage, and adequate nutritional management.
• The effects of these drug treatments also depend on the fistula distance and the amount of discharge.
• Percutaneous intestinal drainage is possible to be very effective in improving skin erosion.

## 1 Introduction

Enterocutaneous fistulas (ECFs) that occur following gastrointestinal surgery require long-term hospitalization, and treatment may be difficult in rare cases [1,2]. Although the morbidity and mortality associated with ECF have decreased over the past 50 years with the advent of novel antibiotics, nutritional support, wound care, the overall mortality is still surprisingly high. Mortality rate has been reported from 5.3 to 30.4% [3,4]. We herein describe a case that we experienced in which we were able to close a refractory enterocutaneous fistula that had occurred following gastrointestinal surgery, by percutaneously puncturing the digestive tract and inserting a catheter in order to drain the digestive fluid. This work is reported in line with the SCARE criteria for case report publication [5].

## 2 Case presentation

### 2.1 Patient: a 79-year-old male

Past medical history: hyperlipidemia, benign prostatic hypertrophy (BPH)

Course of present illness: Laparoscopic sigmoid colectomy was performed for sigmoid colon cancer at another hospital, and a refractory colocutaneous fistula developed due to subsequent suture failure. Because conservative treatment was not successful, he was referred to our hospital to receive colectomy and cutaneous fistula closure at our department. His postoperative course was good, and he subsequently underwent follow-up examinations at our department. However, at his fifth annual postoperative examination, sigmoid colon cancer was detected 10 cm from the previous anastomotic area, requiring surgery. Laparoscopic high anterior resection (HAR) was performed, including resection of the previous anastomotic area.

**Fig. 1:**
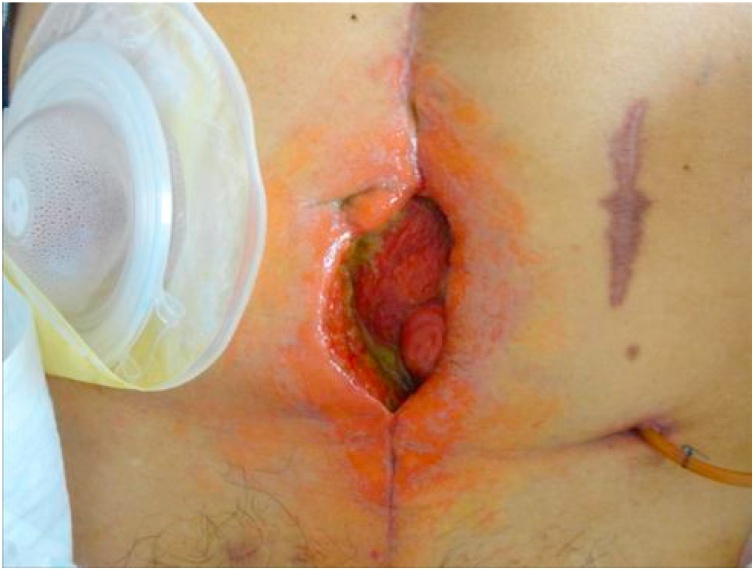
Skin redness and erosion formation.

**Fig. 2:**
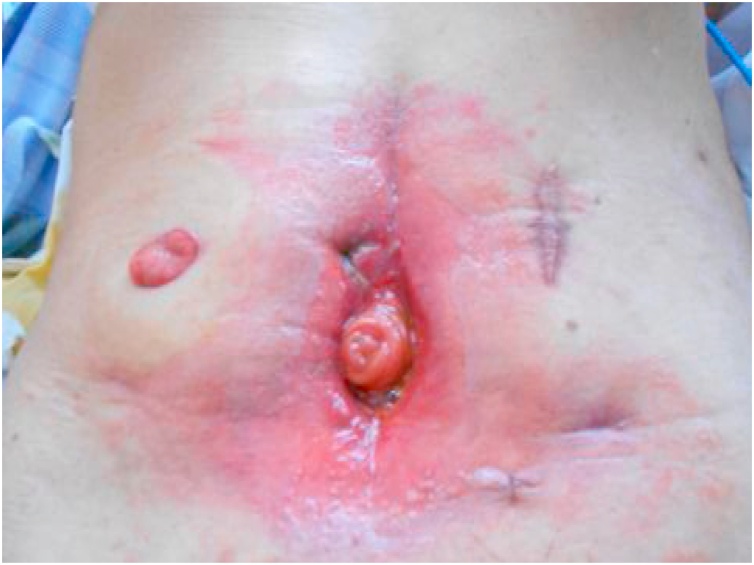
Skin irregularities appear due to granulation.

**Fig. 3:**
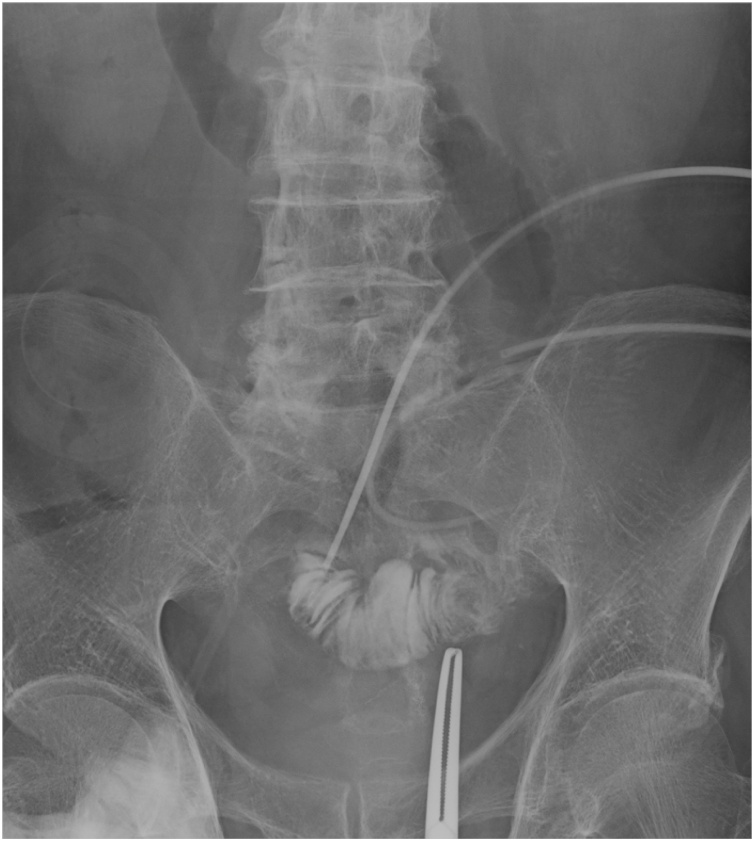
Fluoroscopy.

Postoperative course (1): On the 4th postoperative day, intestinal fluid leaked was drained, and the patient was diagnosed as having suffered suture failure. Because there was no evidence of peritonitis, conservative treatment with fasting and antibacterial drugs was initiated. However, despite undergoing conservative treatment for one month following surgery, no fistula closure was observed. An ileostomy was constructed to allow him to resume oral nutrition. Intraperitoneal adhesions were severe and were removed whenever possible. Starting on the day following surgery, intestinal fluid began to be discharged from the median surgical incision. Apparently a new enterocutaneous fistula (small intestine-skin) had been caused by the surgery. We initially handled the situation by placing a stoma pouch to protect the skin. Several liters of intestinal fluid were discharged each day, and his condition worsened. He then entered the intensive-care unit (ICU) and received intensive care under the ventilator. After leaving the ICU, intestinal fluid continued to be discharged from the median incision. Therefore, negative pressure management (-30 cmH_2_O) was performed on the median incision. The intestinal fluid extensively stimulated the skin, causing redness and the formation of erosions and resulting in a refractory ECF with spontaneous pain (Fig. 1). Although increasing the negative pressure from -30 to -50 cmH_2_O decreased the leakage and temporarily improved the skin erosion and granulation, further skin depression appeared due to the formation of granulation, enabling the pouch to adhere to the skin and worsening the skin symptoms (Fig. 2). Two months after construction of the ileostomy, percutaneous intestinal puncture was performed under fluoroscopy, and contrast medium was injected to confirm the running of the oral intestinal tract (Fig. 3). Percutaneous intestinal drainage was subsequently performed under general anesthesia in the operating room.

**Fig. 4:**
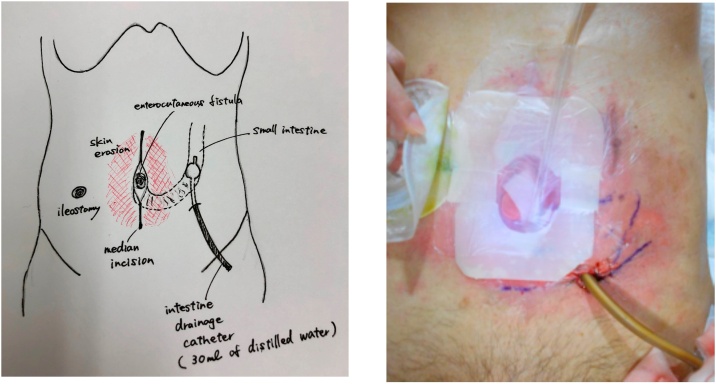
a, b: After percutaneous intestinal drainage.

Surgical findings (percutaneous intestinal drainage): We attempted percutaneous drainage by approaching it away from enterocutaneous fistula. First, a 16-Fr urethral catheter was inserted via the intestinal opening of the skin fistula. Contrast medium was injected in order to confirm the course and position of the small intestine. We made a 3-cm skin incision on the left lower abdomen and sectioned the fascia and peritoneum. The wall of the small intestine was identified immediately below. The intestinal wall was then incised, and the mucosal surface was confirmed. A guide wire was inserted using an 18-G needle as a sheath. It was confirmed that the guidewire had been inserted into the intestinal tract under fluoroscopy. Urinary dilators were inserted in order, starting at 8 Fr, and dilation was performed up to 24 Fr. A 20-Fr urethral catheter (functioning as a drainage catheter, referred to as an intestinale drainage catheter) was inserted into the proximal small intestine and balloon-fixed with 30 mL of distilled water. Contrast medium was injected to confirm that there was no leakage to the anal side. The incised intestinal wall, peritoneal fascia and the skin were closed with sutures, and then the operation was completed (Fig. 4a, b).

### 2.2 Postoperative course (2)

**Fig. 5:**
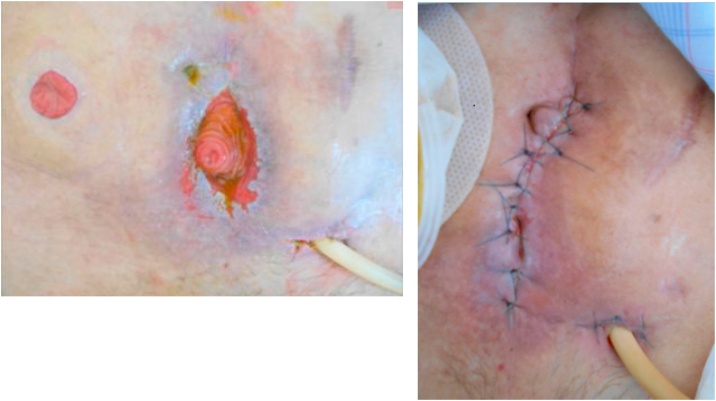
a, b: Erosion improvement and suture closure.

**Fig. 6:**
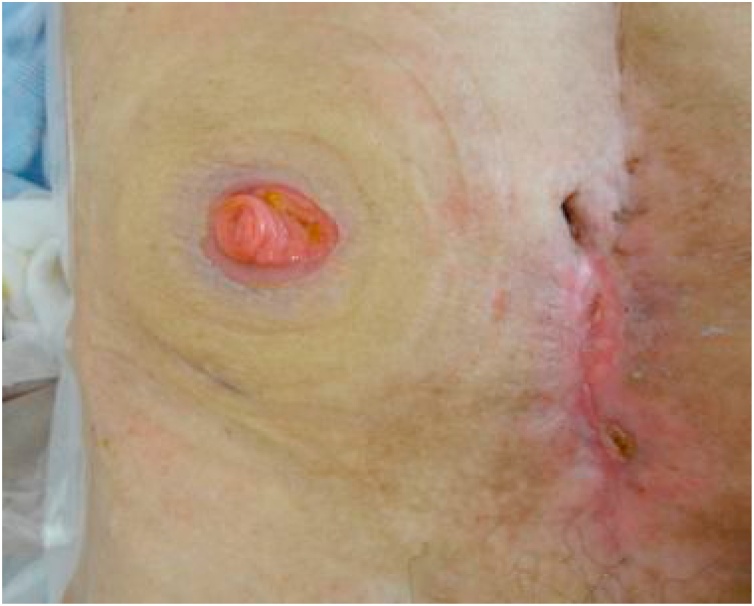
After removing the drainage catheter.

Following surgery, continued negative pressure management (-30 cmH_2_O) was performed for both the median incision and intestinal drainage catheter. This treatment clearly reduced discharged fluid and both the skin redness and erosion gradually improved (Fig. 5a). Four weeks following percutaneous intestinal drainage, the ECF of the median incision was closed with sutures. Both the opened intestinal wall and skin were closed (Fig. 5b). Even after the fistula closure, the intestinal drainage catheter continued to perform continuous suction drainage. The intestinal fluid was passed out from the ileostomy, without any problems in the closed median incision. On the seventh day after closing the fistula, the balloon with the intestinal drainage catheter was deflated (distilled water 30 mL to 15 mL) and the lack of any problems with the closed median incision was confirmed. After meals were resumed, the intestinal drainage catheter was clamped 15 days after closing the fistula and then removed entirely on the 22nd day. No subsequent problems were noted in the abdominal findings (Fig. 6).

## 3 Discussion

ECFs following gastrointestinal surgery occur due to intestinal adhesiolysis and suture failure, even with the development of modern surgical techniques. It is especially likely to occur following peritonitis surgery, multiple laparotomy histories, and post-radiation therapy. The types of fistulas are classified into labial fistulas and tubular fistulas [6]. Labial fistulas have a mucous membrane that can be seen on the surface of the skin like a stoma, and because natural closure cannot be obtained, treatment is very difficult. Tubular leaks are fistulas without mucous membranes on the skin surface and may naturally close as wound healing progresses.

There is no general definition of refractory ECFs, with conservative treatment periods varying; even in the literature describing conservative approaches, the treatment period has varied. One study reported that more than 90 % of cases of natural closure required 4–5 weeks, and another reported that the average period of negative pressure therapy was 4 weeks [7]. Given the above, it seems that four weeks is the breakpoint. The present case was also defined as refractory, as no improvement was noted with conservative treatment for over four weeks. ECFs often occur around the surgical incision, and inflammatory adhesion between the intestine and the surgical incision may be involved in the mechanism. The present patient had a history of three laparotomies, and adhesion was severe due to two instances of suture failure. The digestive fluid did not seem to have spread throughout the abdominal cavity due to the surrounding advanced adhesion and weakening of the abdominal walls, resulting in the formation of an ECF without concurrent peritonitis.

The skin around the refractory ECF is exposed to strong stimulation, due to the digestive fluid containing activated pancreatic juice and bile, delaying closure and causing skin erosion or ulcers, which can inflict significant pain in patients [8].

The basic principles underlying treatment for ECFs are essentially fasting, drainage, and adequate nutritional management [9]. The introduction of total parenteral nutrition (TPN) is commonly effective as aggressive nutrition support. It was previously reported that the development of TPN resulted in an increase in spontaneous closure and reduction in mortality in case of ECFs [[10], [11], [12]]. Furthermore, there have been reports concerning the efficacy of administering antibacterial drugs for infection control, supplementation of blood coagulation factor XIII when it is decreased, and recently, fibrin glue and somatostatin analog injection into the fistula [13,14]. The effects of these treatments also depend on the fistula distance and amount of discharge. Generally, fistulas treated successfully with this method have been low output and short in length [15,16].

**Fig. 7:**
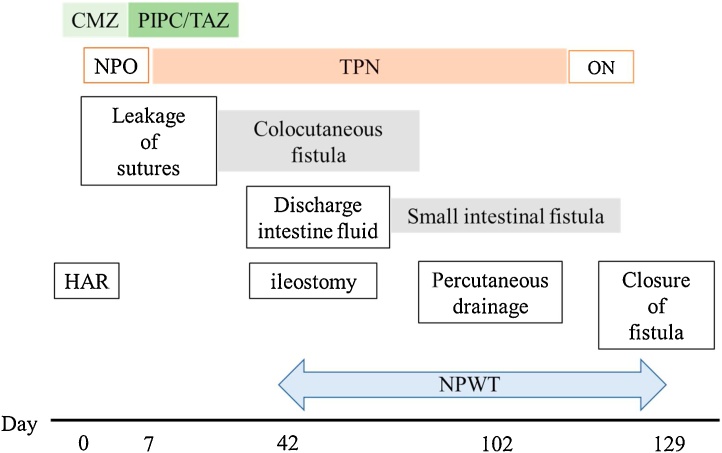
Course of treatment. CMZ: cefmetazole, PIPC/TAZ: piperacillin/tazobactam. NPO: non per oral, TPN: total parenteral nutrition, ON: oral nutrition. HAR: high anterior resection. NPWT: negative pressure wound therapy.

This was a case of a repeated refractory ECF. At the first instance, intestinal resection including the anastomotic area and fistula closure were performed for management of a colocutaneous fistula. The patient developed an ECF the second time, which did not improve after a month of conservative treatment, and an ileostomy was constructed to allow the patient to resume meals; however, as a result, a small intestinal fistula developed. Although it was ultimately considered necessary to perform radical surgery to remove the small intestinal fistula that had formed as a lump together with the fistula skin, the surgery was considered difficult due to the advanced adhesion, and a high risk of postoperative complications and short bowel syndrome; therefore, based on the report of Matsutani et al. [17], percutaneous drainage of the proximal intestine was selected instead (Fig. 7). The keys to successful surgical intervention are making an optimal condition and performing technically precise procedure.

In the present case, although the leakage of digestive fluid from the ECF could not be completely stopped, after an intestinal drainage catheter was inserted and fixed with 30 mL of distilled water, the amount of drainage appreciably decreased, and this approach was very effective in improving the skin erosion, enabling closure with a one-time suture. Furthermore, it was assumed that the continuance of intestine drainage by leaving the catheter following closure of the suture made it possible to maintain the rest of the sutures and thus prevent recurrence of the enterocutaneous fistula.

## 4 Conclusion

Percutaneous intestinal drainage for refractory ECFs following gastrointestinal surgery is minimally invasive and is likely to be extremely useful.

## Declaration of Competing Interest

All authors have no conflicts of interest to disclose concerning the article.

## Funding

All authors have no sponsors to disclose concerning the article.

## Ethical approval

None.

## Consent

Written informed consent was obtained from the patient for publication of this case report and accompanying images.

## Author contribution

Yoshie Hirayama: Conceptualization, Date curation, Writing- Original draft preparation.

Tadashi Koga: Conceptualization, Data curation.

Masanori Kai: Reviewing and Editing.

Kiyoshi Kajiyama: Reviewing and Editing.

## Registration of research studies

1 Name of the registry:
2 Unique identifying number or registration ID:
3 Hyperlink to your specific registration (must be publicly accessible and will be checked):

## Guarantor

Yoshie Hirayama.

## Provenance and peer review

Not commissioned, externally peer reviewed.
